# Event-related potential practice effects on the Paced Auditory Serial Addition Test (PASAT)

**DOI:** 10.2478/v10053-008-0123-z

**Published:** 2012-11-16

**Authors:** Jeffrey M. Rogers, Alison M. Fox

**Affiliations:** 1 School of Psychology, Australian Catholic University, Sydney, NSW; 2 School of Psychology, University of Western Australia, Perth, WA

**Keywords:** PASAT, event-related potentials, practice-effects, attention

## Abstract

Practice can change the nature and quality of a stimulus-response relationship. The current study observed the effects of repeated administration of the Paced Auditory Serial Addition Test (PASAT) in 12 healthy individuals, in an effort to establish distinct profiles associated with novel and practiced processing. Over four training sessions the mean number of correct responses on this demanding test of attention significantly improved and was approaching ceiling for most task conditions. Behavioural improvements were associated with significantly reduced amplitude of late Processing Negativity, a frontally distributed component of the event-related potential waveform associated with voluntary, limited-capacity activity within higher-order attentional systems. These results suggest that PASAT performance became more efficient as practice seemingly eased the strategic planning and coordination requirements the task places on frontally-mediated executive attention resources. The findings of the current study extend our understanding of the functional and behavioural mechanisms underlying the effects of practice.

## Introduction

The Paced Auditory Serial Addition Test (PASAT; The Psychological Corporation, 1998) is one of the most frequently used tests to evaluate attentional functioning (Tombaugh, 2006). The task was first developed by Gronwall and Sampson (1974) to examine the effects of head injury on information processing, and over time the PASAT has been shown to be sensitive to the neurocognitive effects of many clinical conditions, including multiple sclerosis (Rao & Cognitive Function Study Group of the National Multiple Sclerosis Society, 1990), schizophrenia (Rund et al., 2006), HIV infection (Heaton et al., 1995), and adult ADHD (White, Hutchens, & Lubar, 2005).

The PASAT involves continuous auditory presentation of a random sequence of 61 digits from 1 through 9. Examinees are required to calculate the sum of the most recently presented digit and the digit presented immediately prior, while simultaneously at-tending to the next digit in the series. Thus, the second digit is added to the first, the third to the second, and so on. Each trial of the task therefore requires the examine to register sensory input, retrieve a stored stimulus, perform a mental calculation, respond verbally, and inhibit encoding of their response while attending to the next stimulus in the series, all at an externally determined pace.

The rate at which digits are presented can be adjusted by manipulating the inter-stimulus interval (ISI). Progressively shorter ISIs were originally assumed to place greater demands on information processing speed, thereby increasing task difficulty (Gronwall & Wrightson, 1974). However, PASAT performance has not been found to correlate with measures of simple reaction time (Sherman, Strauss, & Spellacy, 1997), and researchers have routinely failed to identify a reliable relationship between ISI and task sensitivity (Tombaugh, 2006).

Interpretation of the PASAT has therefore been revised. While the multi-factorial nature of the task places demands on a number of cognitive abilities, including processing speed, successful performance ultimately lies in the efficient coordination of the various sub-tasks into one single integrated activity (Cicerone, 2002; Diehr, Heaton, Miller, & Grant, 1998; Spikman, Henk, Deelman, & van Zomeren, 2001). These higher-order strategic planning aspects are the responsibility of executive attention processes, alternatively referred to as *controlled processing* (Shiffrin & Schneider, 1977), the *central executive* (Baddeley & Hitch, 1974), or *supervisory attentional control* (Norman & Shallice, 1986).

Positron emission tomography and functional magnetic resonance imaging studies have supported this reconceptualization of the PASAT (Au Duong et al., 2005; Audoin et al., 2005; Forn et al., 2006; Lockwood, Linn, Szymanski, Coad, & Wack, 2004). Successful PASAT performance has been shown to be dependent upon activation within the anterior cingulate gyrus, left pre-frontal, superior temporal, and parietal lobes. Activation of the cingulate cortex has been regarded as representing involvement of the anterior attention network (Posner & Petersen, 1990) mediating higher-order attentional functioning (Au Duong et al., 2005; Audoin et al., 2005; Lockwood et al., 2004). Superior temporal lobe activation was associated with auditory perceptual and mnestic processes (Au Duong et al., 2005; Audoin et al., 2005; Lockwood et al., 2004) while frontal and parietal lobe activation was associated with working memory and executive attention (Au Duong et al., 2005; Audoin et al., 2005; Forn et al., 2006; Lockwood et al., 2004). Interestingly, analysis of the activation patterns failed to identify a specific math-related neural site (e.g., intraparietal sulcus; Dehaene, Piazza, Pinel, & Cohen, 2003), suggesting that the addition of 2 one-digit numbers is a fairly basic task that proceeds relatively automatically (Lockwood et al., 2004).

### PASAT practice effects

Serial administration of neuropsychological tests is a common method in research and clinical practice for longitudinal assessment of function. A number of processes can contribute to changes over time in neuropsychological test performance, including progression of an underlying disease, recovery from neurological insult, or manipulation of an independent variable. However, a practice effect, the learning which results from repeated task exposure, can also contribute and frequently confounds the interpretation of change over time.

Significant practice effects have been observed on the PASAT. When tested on two occasions 1 week apart, healthy controls performed about 6 points higher at re-test (Stuss, Stethem, & Poirier, 1987). Gronwall (1977) contended practice effects were minimal after the second administration; however, others have reported continued improvement over three (Beglinger et al., 2005), four (Beglinger et al., 2005; Stuss, Stethem, Hugenholtz, & Richard, 1989), and even eight sessions (Beglinger et al., 2005; Feinstein, Brown, & Ron, 1994; Stuss et al., 1989). Of note, no association has been reported between the length of the test-retest interval and magnitude of the practice effect. Baird, Tombaugh, and Francis (2007) reported comparable improvement in performance between groups retested after 20 min, 1 week, or 3 months.

Practice effects are presumably based on the enhanced operation of cognitive mechanisms which were first activated during initial novel processing (Jansma, Ramsey, Slagter, & Kahn, 2001; Miller, 2000). The particular improvement in performance over repeated administrations of the PASAT has been hypothesized to be due to an active learning process directing the strategic processing of target stimuli and suppression of distracting stimuli (Feinstein et al., 1994; Spikman, Timmerman, van Zomeren, & Deelman, 1999; Tombaugh, 2006). However, how the executive attention processes underlying PASAT performance are affected by practice has not yet been directly explored.

### Event-related potentials

Event-related potentials (ERPs) provide a non-invasive neurophysio-logical index of sensory processing and mental functions (Connolly, D‘Arcy, Newman, & Kemps, 2002; Solbakk, Reinvang, & Nielsen, 2000). Due to their high temporal resolution, ERPs can provide unique information about the neural processes that mediate information processing (e.g., Andreassi, 1995; Rugg & Coles, 1995).

The Processing Negativity (PN) component of ERP waveforms is generally considered an electrophysiological marker of focused, sustained auditory attention (Näätänen, 1982). PN is a slow, negative waveform composed of separate early and late peaks (Näätänen, 1982). The early PN component is generated in modality-specific sensory are-as and appears to reflect initial stimulus selection (Solbakk, Reinvang, Nielsen, & Sundet, 1999). Late PN is a frontally distributed component, believed to reflect voluntary, limited-capacity executive attention or working memory activity responsible for coordinating and maintaining goal-directed stimulus processing (Näätänen, 1982, 1985; Potter & Barrett, 1999; Solbakk et al., 1999). Historically, PN has been investigated using a dichotic listening task and subtracting the ERPs of the unattended channel from the attended channel. However, Näätänen (1982) speculated that this subtraction method is not necessarily ideal for obtaining PN, and Potter and Barrett (1999) argued that late PN can be directly observed in the waveform elicited by a single-channel of attended stimuli.

Research exploring the effects of repeated auditory attention task testing on the late PN component has provided a robust effect. Over a series of six (Shelley et al., 1991) and seven (Woods, 1990) training sessions on experimental “oddball” tasks, late PN component amplitude was significantly reduced. In each study, amplitude attenuation was accompanied by increases in task accuracy.

### Aims of the current study

The purpose of the current experiment was to re-examine the serial performance of healthy individuals on the PASAT to identify possible behavioural and electrophysiological markers of novel and practised information processing. Such an investigation could improve our understanding of the practice effects which occur over repeated presentation of the PASAT, particularly the role of executive attention as performance becomes more efficient and effective.

Previous studies have identified behavioural profiles which reliably distinguish between novel and practiced information processing (Jansma et al., 2001; Logan & Klapp, 1991; Shiffrin & Schneider, 1977). Following consistent practice of the general PASAT procedure, performance was therefore expected to become faster, more accurate, and less variable.

However, behavioural measures reflect the end products of multiple underlying cognitive operations and provide limited insight into on-going processing (Birnboim, Breznitz, Pratt, & Aharon, 2002). Functional neuroimaging studies have demonstrated that consistent practice is accompanied by diminished activation within frontally mediated executive attention regions (Jansma et al., 2001; Raichle et al., 1994). Similarly, Shelly and colleagues (1991) and Woods (1990) found late PN amplitude attenuation following repeated task administration. PASAT behavioural practice effects in the current study were therefore expected to be associated with a reduction in the amplitude of the late PN component of the ERP waveform. The contribution of other cognitive ERP components to novel and practiced PASAT performance was also explored.

## Methods

### Participants

Twelve healthy individuals responding to a general university advertisement participated in this experiment. There was no reimbursement for participation. The participant group consisted of four right-handed and two left-handed males, and six right-handed females. All denied a history of head injury, psychiatric condition, neurological disorder, epilepsy, or drug abuse. Participants had normal hearing and normal or corrected to normal vision. The mean age of the participants was 26.5 years (*SD* = 6.9 years, range 17-37 years). This research was approved by the Human Research Ethics Committee of the University of Western Australia, and each participant provided written informed consent for voluntary participation.

### Task

Participants were administered a computerized, children’s version of the PASAT (The Psychological Corporation, 1998). The children’s version of the task presents numbers from 1 to 9 but minimizes the possible confounding effect of mathematical ability on performance by using sums that do not exceed 10 (Royan, Tombaugh, Rees, & Francis, 2004). A trial consisted of 61 digits (60 sums), and testing sessions included one trial at each of four ISI rates (2.4, 2.0, 1.6, and 1.2 s). Digits were presented via headphones, and spoken responses were registered via a microphone and digital recorder. Vocal responses were automatically digitized and recorded for off-line analysis.

### Procedure

Participants attended four sessions over a mean 24 day period (*SD* = 13.0 days, range 4-47 days; longer delays related to equipment malfunction). During each experimental session participants were administered one PASAT trial at each of the four ISIs, in randomized order. Participants were asked to minimize eye and body movements and speak quietly when responding. The total number of correct responses on each trial was used to quantify performance accuracy (max = 60). Two-way repeated measures ANOVAs were conducted to compare the mean number of correct PASAT responses at each experimental session and ISI. Where Mauchly’s test of sphericity was violated, Greenhouse-Geisser corrections were applied.

On the first and fourth experimental sessions, continuous electroencephalogram (EEG) activity was recorded during PASAT performance. ERP component amplitudes and latencies were analysed in a two-way repeated measures ANOVA with Session (one and four) and ISI (2.4, 2.0, 1.6, and 1.2 s) as within-subjects factors. Greenhouse-Geisser corrections were again applied as necessary.

Participants also completed a control version of the PASAT during the first and fourth sessions. In this version, referred to as the speech task, participants were administered a single trial at the 1.6-s ISI and requested to overtly repeat each presented digit, rather than perform the standard task. During the speech task ERPs were simultaneously recorded.

### EEG data acquisition and analysis

Continuous EEG recordings were obtained using a 32 tin electrode Neuroscan QuikCap. Vertical and horizontal electro-culograms were recorded with electrodes placed at the supraorbital ridge and suborbital region of the left eye, and at the outer canthus of each eye. All electrodes were referenced to nose electrodes, and impedances were adjusted to below 6 k. EEG activity was sampled continuously at a digitization rate of 250 Hz. The signals were amplified with a Neuroscan Incorporated (Herndon, VA) SynAmps system with a band-pass filter of 0.05-30 Hz (-6 dB down). SCAN 4.0 software was used to register and analyse EEG activity. Prior to averaging, statistical eye movement correction was performed offline using the principal components transform included in the SCAN software. After baseline adjustment around a 100 ms pre-stimulus interval, remaining epochs containing amplitudes in excess of 75 V at any electrode were rejected. Stimulus-locked cognitive ERPs were then averaged over 1 s epochs, including a 100 ms pre-stimulus baseline period and a 900 ms post-stimulus period.

The following criteria were derived from visual inspection of the grand-averaged waveforms and used in the statistical analysis of the ERP components of interest: *N1* was defined as the most negative peak in the interval 80-160 ms, *P2* as the most positive peak in the interval 160-290 ms, *N2* as the most negative peak in the interval 280-340 ms, *P3* as the most positive peak in the interval 450-780 ms,and *late PN* was defined as the mean negativity in the interval 380-650 ms. Visual inspection of the grand-averaged waveforms was also used to determine the electrode site over which each ERP component reached maximum activation. Mean amplitude (V) and peak latency (milliseconds) of each component for each participant were then calculated from this electrode site. The amplitude and latency of the N1 and P2 components were measured at the central scalp site, Cz. P3 was measured at the parieto-central site, Pz. The N2 and late PN components were measured at the fronto-central site, Fz.

## Results

### Behavioural data

Mean correct PASAT responses at each session and ISI are presented in Table 1. Participants demonstrated improvement in the number of correct PASAT responses over time, *F*(3, 33) = 26.51, *p* < .01, η_p_^2^ = .71.Bonferroni corrected (*p* < .0167) paired-samples t-tests indicated that the improvements were statistically significant from the first to the second experimental session.

**Table 1.** Mean Correct PASAT (The Paced Auditory Serial Addition Test) Responses at Each Experimental Session Across the Four ISIs

	ISI			
	2,4 s	2,0 s	1,6 s	1,2 s
Session 1	54,3 (3,7)	51,9 (4,6)	49,6 (8,9)	40,1 (10,0)
Session 2	57,3 (2,8)	57,1 (3,5)	53,1 (6,1)	46,3 (10,8)
Session 3	56,9 (3,1)	56,6 (4,6)	55,4 (6,4)	49,8 (11,2)
Session 4	58,5 (1,6)	58,4 (1,8)	56,9 (2,5)	50,8 (8,6)

*Note*. Maximum possible correct = 60. ISI = the inter-stimulus interval. Standard deviations in parentheses.

The mean number of correct PASAT responses improved with increasing ISI, *F*(1.13, 12.37) = 16.46, *p* < .01, η_p_^2^ = .60, with post-hoc t-tests indicating that at each experimental session the participants produced a significantly reduced mean number of correct responses at the 1.2-s ISI condition.

**Figure 1.**
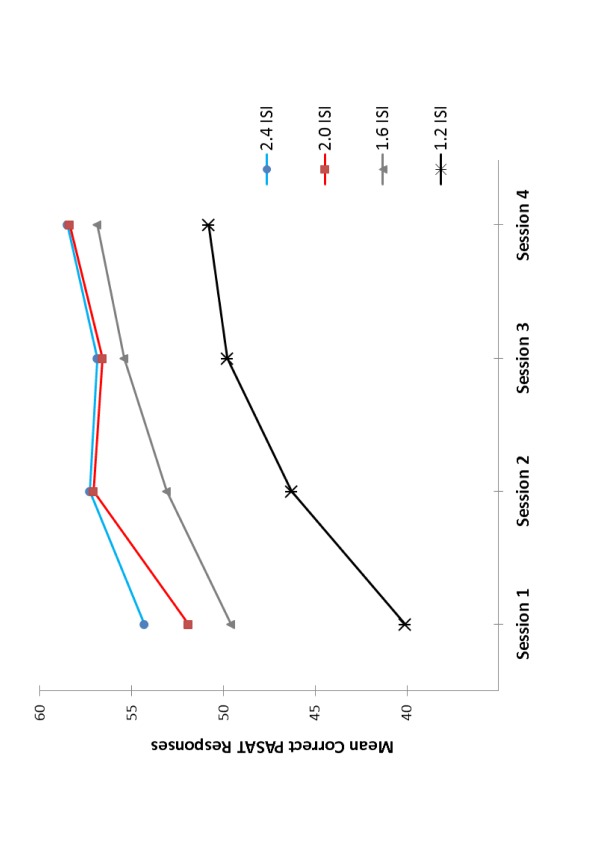
Mean correct PASAT (The Paced Auditory Serial Addition Test) responses at each experimental session across the four ISIs (inter-stimulus intervals).

Finally, the interaction between Session and ISI was also significant, *F*(3.94, 43.32) = 2.75, *p* = .04, η_p_^2^ = .20. As can be seen in Figure 1, over time PASAT performance became more accurate and less sensitive to ISI manipulations. In particular, the difference in mean correct responses between the two most extreme ISIs, 2.4- and 1.2-s, significantly decreased from 27% in the first to 13% in the fourth session, *t*(11) = 6.65, *p* < .01. Correspondingly, the increase in accuracy from the first to the fourth session was larger for the 1.2-s ISI than for the 2.4-s ISI(22% vs. 7%), *t*(11) = 6.21, *p* < .01.

Previous research (e.g., Wigenfeld, Holdwick, Davis, & Hunter, 1999) has commented on the lack of reliability of the reaction time data provided by the PASAT computerized software. Although the program allows the examiner to manually calibrate the signal detection threshold, the calculated reaction time still does not often coincide with the onset of the vocal response. Rather, throat clearing or sighs which preceded the scorable response were registered. Due to the unreliability of the measurement system, PASAT reaction time data were considered exploitative only. However, as expected, reaction times in Table 2. quickened over sessions, *F*(3, 33) = 6.81, *p* < .01, η_p_^2^ = .38, and with decreasing ISI, *F*(1.63, 17.97) = 16.71, *p* < .01, η_p_^2^ = .60.

**Table 2.** Mean Correct PASAT (The Paced Auditory Serial Addition Test) Reaction Time at Each Experimental Session Across the Four ISIs

	ISI			
	2,4 s	2,0 s	1,6 s	1,2 s
Session 1	0,8 (0,3)	0,8 (0,2)	0,7 (0,1)	0,6 (0,1)
Session 2	0,8 (0,2)	0,8 (0,2)	0,7 (0,2)	0,6 (0,1)
Session 3	0,8 (0,3)	0,7 (0,2)	0,7 (0,2)	0,6 (0,2)
Session 4	0,6 (0,2)	0,7 (0,2)	0,6 (0,2)	0,5 (0,1)

*Note*. Reaction time in seconds. ISI = the inter-stimulus interval. Standard deviations in parentheses.

### ERP results

**Figure 2.**
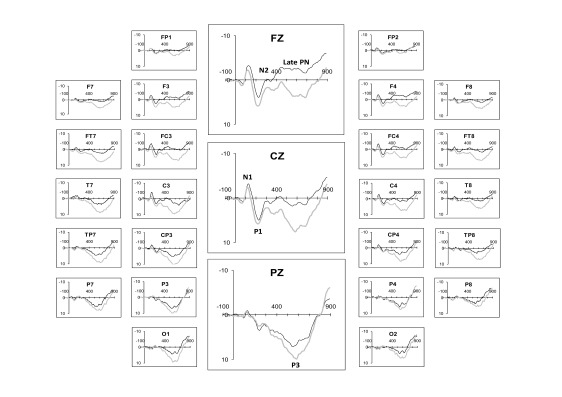
Grand averaged ERPs elicited in response to PASAT (The Paced Auditory Serial Addition Test) stimuli during Session 1 (thin line) and Session 4 (thick line).

**Figure 3.**
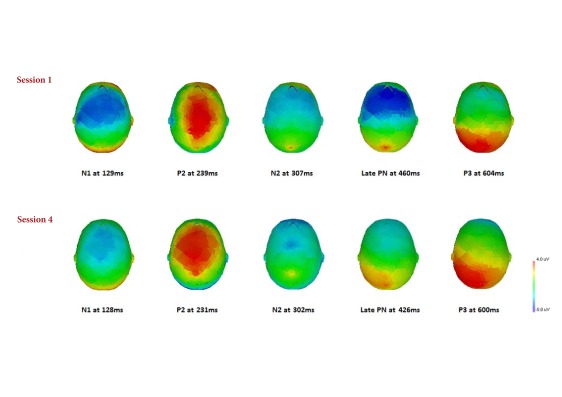
Session 1 and 4 topographical maps of the event-related potential components at peak amplitude during PASAT (The Paced Auditory Serial Addition Test) performance.

Grand-averaged responses from the first and fourth experimental session are presented in Table 3. N1, P2, N2, and P3 components were apparent at both sessions. During Session 1, a frontally distributed negativity (late PN), was observed at approximately 400 ms but at Session 4 there was a marked attenuation of this component (see Figures 2 and 3).

**Table 3.** Mean Amplitude and Peak Latency Data Across Experimental Sessions for the Cognitive Event-Related Potential Components Elicited During Correct PASAT (The Paced Auditory Serial Addition Test) Responses

Component			M	SD
N1	Session 1	Amplitude	-3,2 μV	0,8 μV
		Latency	129 ms	11 ms
P2	Session 1	Amplitude	5,0 μV	1,1 μV
		Latency	239 ms	21 ms
N2	Session 1	Amplitude	-0,2 μV	1,7 μV
		Latency	307 ms	9 ms
P3	Session 1	Amplitude	7,1 μV	2,3 μV
		Latency	604 ms	16 ms
Late PN	Session 1	Amplitude	-2,9 μV	2,2 μV
		Latency	460 ms	13 ms
Component			M	SD
N1	Session 4	Amplitude	-2,4 μV	0,8 μV
		Latency	128 ms	13 ms
P2	Session 4	Amplitude	5,8 μV	1,2 μV
		Latency	231 ms	20 ms
N2	Session 4	Amplitude	1,7 μV	1,4 μV
		Latency	302 ms	13 ms
P3	Session 4	Amplitude	11,7 μV	2,2 μV
		Latency	600 ms	19 ms
Late PN	Session 4	Amplitude	0,4 μV	1,8 μV
		Latency	426 ms	13 ms

Two-way repeated measures ANOVAs revealed there was no significant within-group effect of ISI or session on the latency of any ERP component. Furthermore, neither ISI nor session had a significant effect on the amplitude of the N1, P2, N2, or P3 components. The mean amplitude of the late PN component was significantly reduced following practice, *F*(1, 11) = 6.63, *p* = .03, η_p_^2^ = .38, and was not significantly affected by ISI manipulations, *F*(3, 33) = 0.27, *p* = .85, η_p_^2^ = .02.

**Figure 4.**
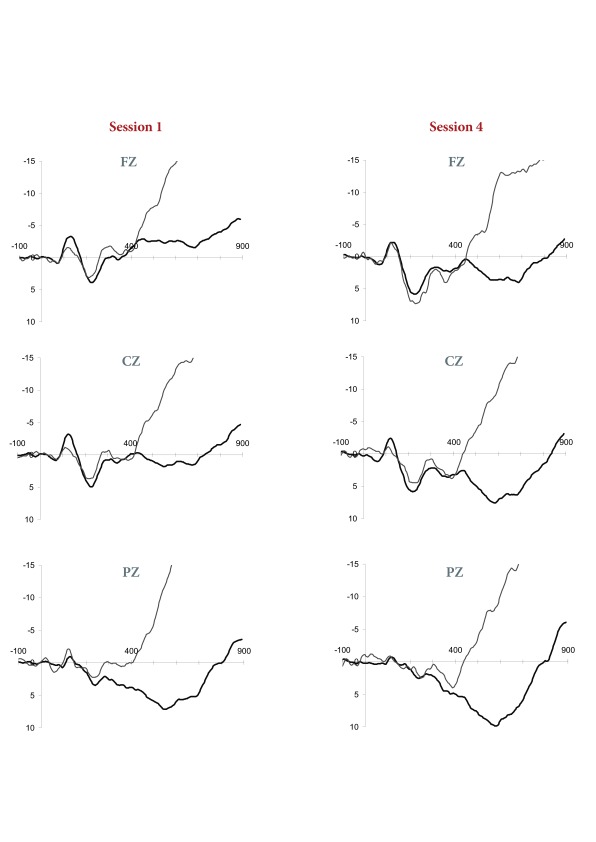
Session 1 and Session 4 PASAT (The Paced Auditory Serial Addition Test) versus speech task (thick vs. thin line, respectively) grand averaged responses.

To investigate the effects of speech upon the results, ERPs generated in the speech task were compared against grand-averaged waveforms generated during correct PASAT responses, collapsed across ISIs. As can be seen in Figures 4, the speech waveforms were characterized by early fronto-centrally distributed sensory evoked potentials. Across both sessions, beginning at approximately 400 ms, and corresponding with the speech response, a topographically non-specific negative deflection emerged and rapidly increased over the remainder of the epoch.

Re-examination of the PASAT grand-averaged waveforms revealed a similar topographically non-specific, rapidly increasing negativity. However, the onset of this negativity is greatly delayed in the ERPs generated during performance of the PASAT task, beginning at approximately 700 ms (corresponding with the PASAT reaction time data), and following the late cognitive ERP components.

## Discussion

The PASAT is a challenging task which requires multiple, competing information processing demands to be flexibly combined to adhere to a novel set of task contingencies. Executive attention processes are considered critical for dynamically establishing and coordinating the multiple sub-skills underlying successful task performance. With repeated administration, PASAT performance is known to reliably improve. Although widely reported, this practice effect is poorly understood.

In general, practice effects have been attributed to an easing of task demands related to the extended training of a consistent set of processing operations. The connectionist/control model of information processing argues information is processed and stored within a network of excitatory and inhibitory neural nodes, which are organized into networks of increasingly complex input and output connections (Schneider & Chein, 2003; Schneider & Detweiler, 1988). Knowledge is not stored, represented, or manipulated in any specific node of a network, but in the pattern of interconnections of any number of nodes and the connectionist weights between the nodes (Schneider, 1987). Practice can change the pattern and strength of connections, and hence the output or efficiency of a network, as associations within and between information processing networks are strengthened through repeated activation.

In connectionism, frontally-mediated, capacity-limited attentional control is responsible for the deliberate activation, maintenance, or enhancement of information processing nodes, and strategic suppression of unwanted activation (Schneider & Chein, 2003; Schneider & Detweiler, 1988). Following consistent practice, always requiring the same sequence of processing operations, associative connections between information processing nodes strengthen such that input can evoke the appropriate output pattern independent of executive attention (Schneider & Detweiler, 1988).

In contrast, Logan (1988; Logan & Klapp, 1991) has proposed an “instance theory” to explain the mechanism through which information processing improves with practice. According to this theory, improvement occurs with the acquisition of a domain-specific knowledge base formed of separate representations termed instances.

Processing of tasks is effortful at first and utilizes domain-general processes of knowledge-retrieval, which Logan (1988) refers to as algorithms. However, attending to a stimulus also encodes an instance in memory of how that stimulus was processed. Each encounter with a stimulus is encoded, stored, and retrieved separately, and repeated exposure to a stimulus will increase the number of stored instances of how that stimulus was processed. With practice, performance of a task becomes more and more efficient because it is quicker and easier to automatically retrieve past instances of successful processing solutions rather than consciously construct an algorithm.

Both theories propose the positive effect of practice relies on the efficient retrieval of knowledge, whether this efficiency is conceptualized by the strength of associations or the number of instances. The current study has demonstrated that four experimental sessions of practice facilitate change in the processing efficiency underlying successful PASAT performance in healthy individuals. Following repeated task exposure, participants’ accuracy improved and was less sensitive to the effect of ISI reductions. Concurrent attenuation of the late PN component of the ERP waveform suggested that practiced PASAT performance placed fewer demands on executive attention resources.

### Behavioural data

As expected, participants demonstrated significant improvement from experimental Sessions 1 to 2 in the mean number of correct PASAT responses. While participants continued to improve in the mean number of correct PASAT responses over further sessions, the differences failed to reach statistical significance. These findings are consistent with the PASAT literature, which reports the greatest practice effects occur between the first and second administrations, with smaller improvements thereafter (Beglinger et al., 2005; Gronwall, 1977; Gronwall & Sampson, 1974; Stuss et al., 1989). Particularly at the longer ISIs, a ceiling effect likely contributed to the failure of subsequent administrations in the current study to generate significant improvement (Beglinger et al., 2005).

These results indicate that healthy individuals can rapidly acquire information processing strategies that facilitate more effective and efficient task performance. However, at each session the mean number of correct PASAT responses at the 1.2-s ISI condition remained significantly poorer relative to the remainder of PASAT conditions. Similarly, over two test sessions, Stuss and colleagues (1987) found performance in healthy individuals at the 1.2-s ISI differed significantly from the 2.4-,2.0-, and 1.6-s ISI presentation rates. Why examinees in the current study demonstrated disparate performance trajectories with practice of the 1.2-s ISI condition remains unresolved. Instance theory suggests that after four sessions of practice on the 1.2-s ISI condition, the “race” between effortful, consciously directed algorithms and automatic memory search for an appropriate “instance” was still being routinely won by the algorithm. However it is unclear why the slower algorithm process would persist and prevail at only this most rapid presentation rate.

Alternatively, connectionism theory suggests that the pace of the 1.2-s ISI condition exceeds the efficiency with which information is integrated and sub-routines can be coordinated for the execution of task demands. The most rapid PASAT presentation rate may be impairing the formation and strengthening of associations, prolonging reliance on frontally-mediated control processes. However, to more fully characterize the executive attentional requirements of successful PASAT performance following practice, the ERP data must be examined.

### ERP data

Novel PASAT performance was associated with a late PN deflection of the ERP waveform. Potter and Barrett (1999) also found that processing of the PASAT evoked this frontally distributed negativity in healthy controls. Notably, the amplitude of this deflection was significantly attenuated following repeated PASAT practice. The late PN component has been linked to the central executive of working memory and supervisory attentional systems being engaged in the processing of novel or complex task demands (Hansen & Hillyard, 1988; Näätänen, 1985; Potter & Barrett, 1999). Extended training seemingly eased the strategic planning and coordination requirements the PASAT places on frontally-mediated executive attention resources. Considered with previous serial ERP studies of focussed attention (Shelley et al., 1991; Woods, 1990), the reduction in late PN amplitude observed in the current study provides convergent psychophysiological evidence of the decreased reliance on executive attention resources following consistent practice.

In the current study, neither the amplitude nor the latency of the N1 and P2 components were significantly affected by extended practice. These findings suggest that consistent training had little or no effect on the early sensory processes involved in stimulus registration and discrimination of PASAT stimuli.

No specific hypothesis regarding the N2 component was formulated prior to testing. Visual inspection of the ERP waveforms suggested the amplitude of the N2 component was attenuated following extended practice, but the difference was not statistically significant. This trend may reflect a minor decrease in component amplitude, or could have resulted from a reduction in overlap with frontal negativity as the amplitude of the late PN component decreased over time.

Visual inspection of the ERP waveforms also suggested P3 amplitude increased following consistent practice, although again the difference did not reach statistical significance. Increased P3 amplitude following practice has been suggested to reflect less overlap with late PN (Wijers, Otten, Feenstra, Mulder, & Mulder, 1989) or a non-specific attentional response (Kramer & Spinks, 1991). Additional investigations have demonstrated that practiced performance is characterized by reduced P3 latencies (Hoffman, Simons, & Houck, 1983). In the current study P3 latency remained relatively constant over repeated PASAT administration. The absence of an effect of extended training on the latency of the P3 component may be due to the emphasis the task places on specific stages of information processing (Karayanidis & Michie, 1996; McCarthy & Donchin, 1981). In particular, Spikman, van der Naalt, van Weerden, and van Zomeren (2004) contend that for successful PASAT performance, stimuli must be processed through complex task-specific, rule-based processes. Attention resources are therefore directed primarily toward strategic planning aspects of information processing rather than stimulus-elicited processing reflected in the P3 component. PASAT practice effects are therefore most apparent through examination of the late PN component.

**Figure 5.**
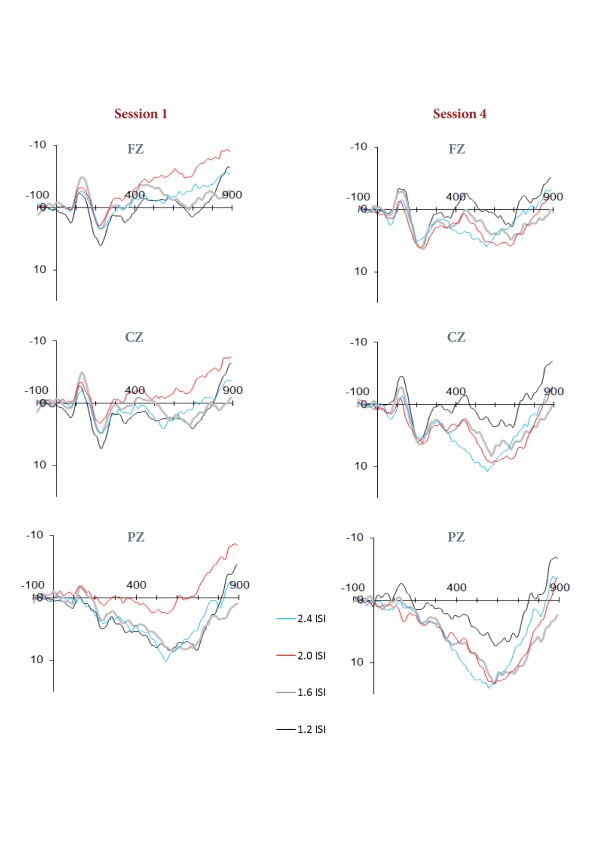
Session 1 and Session 4 event-related potentials elicited in response to PASAT (The Paced Auditory Serial Addition Test) stimuli at each of the four ISIs (inter-stimulus intervals)

In the current study the ERP data at each session was collapsed across ISIs, as there was no significant main effect of ISI or Session × ISI interaction on any of the ERP components of interest. Within a single session of PASAT testing, Potter and Barrett (1999) also found the amplitude of the late frontal negativity component was not responsive to their manipulations of the ISI. However, visual inspection of ERP waveforms generated separately at each of the four ISIs did suggest that following consistent practice, PASAT performance on the 1.2-s ISI condition may have continued to elicit some late PN deflection (see Figures 5). Such a trend would be in keeping with the behavioural differences in mean correct responses observed on the 1.2-s ISI condition relative to the remainder of PASAT ISI conditions following consistent practice.

### Limitations of the current study

The current study utilised a sample of 12 participants. However, each PASAT trial was based on 60 items, providing a robust number of task iterations. Furthermore, the partial eta squared values (reported because the study design used non-independent repeated measures) for the key behavioural and ERP results suggest a magnitude of effect, independent of sample size. However, replication in a larger sample would strengthen the findings of the current study and allow further investigation of the observed trends over time in P3 amplitude, and late PN amplitude on the 1.2-s ISI condition.

Furthermore, the current study included two left-handed clients. Although there was no expected or obtained evidence to suggest lateralisation of any of the processes under investigation, duplication in independent samples, including more left-handed participants, would strengthen the current results.

Finally, both sessions of ERP recordings contained accompanying speech artefact, manifest in the grand-averaged session waveforms as a global negative deflection emerging roughly 700 ms post-stimulus. In their ERP investigation of PASAT performance, Potter and Barrett (1999) also reported a large deflection late in the recording epoch which they attributed to speech artefact. They argued the activation prior to onset of the speech deflection was reasonable to treat as representative of cortical activity. Additionally, in the current study, the major ERP finding was of a *reduction* in late frontal negativity, in spite of persistent speech-related cortical negativity. Regardless, the potential for speech artefact masking or contaminating the ERP results cannot be overlooked.

### Conclusions

There is a vast literature establishing a connection between the frontal lobes and executive functioning (Luria, 1980; Shallice & Burgess, 1991; Stuss & Benson, 1986). The current results extend our understanding of the temporal dynamics of activation within this network responsible for the executive control of attention, and the functional adaptations which take place following consistent practice. Such knowledge may be of particular benefit in interpreting serial performance on the PASAT and other tests of executive function in both healthy and clinical populations.
